# Supplementary material for: Rosavin Ameliorates Hepatic Inflammation and Fibrosis in the NASH Rat Model via Targeting Hepatic Cell Death
Source: Int J Mol Sci. 2022 Sep 5;23(17):10148. doi: 10.3390/ijms231710148 (PMC9456245; doi:10.3390/ijms231710148)
Supplement: Supplementary file 1 [file ijms-23-10148-s001.zip › ijms-1887233-supplementary.pdf]

Supplementary information:

S1 Figure: Validation of the relation between HSPD1, MMP14, ITGB1 & TNF genes to the inflammatory response, oxidative stress, apoptotic process, or cell adhesion using GeneCards database (https://www.genecards.org/)

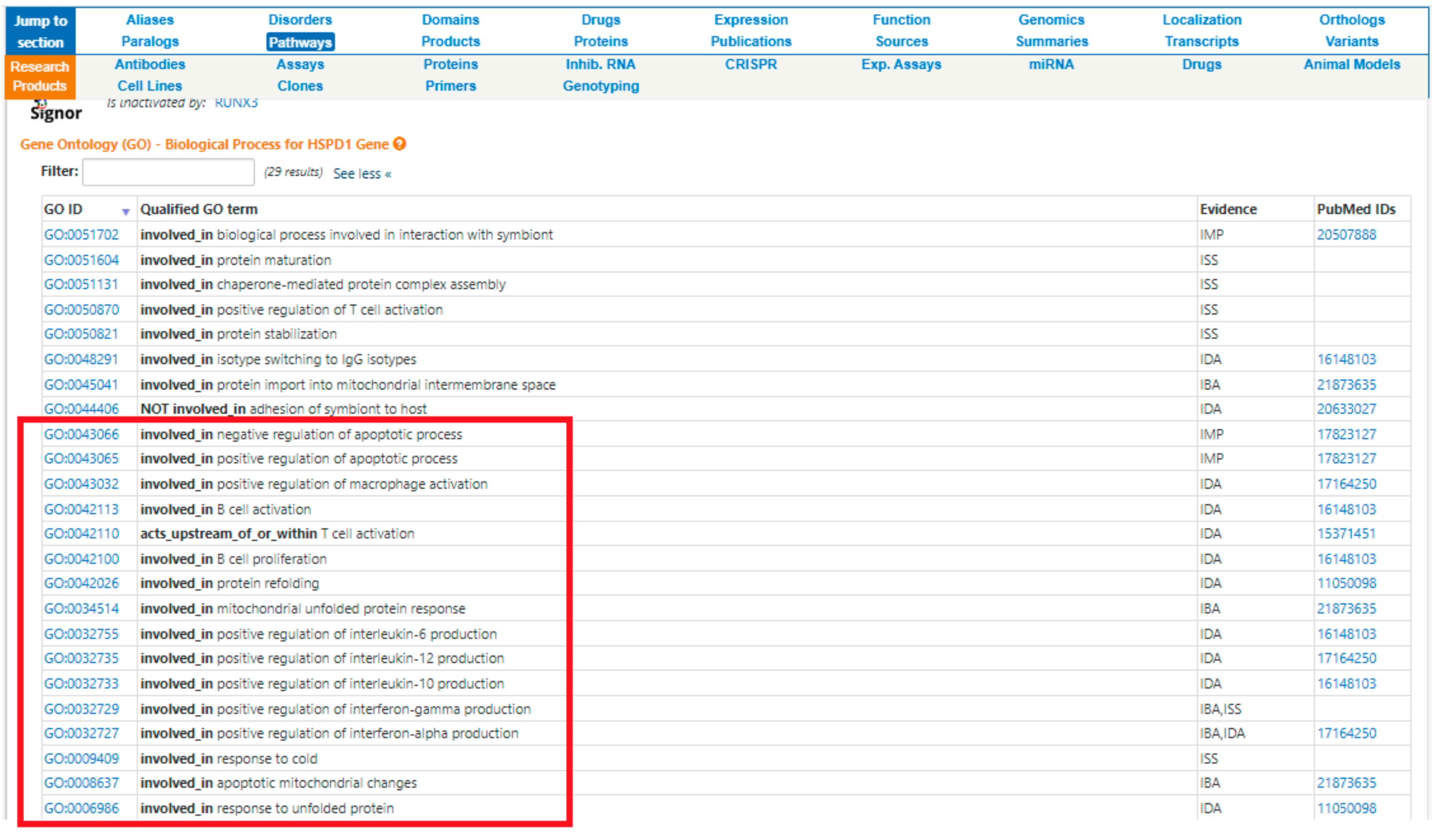

GO ID	Qualified GO term	Evidence	PubMed IDs
GO:0051702	**involved_in** biological process involved in interaction with symbiont	IMP	20507888
GO:0051604	**involved_in** protein maturation	ISS	
GO:0051131	**involved_in** chaperone-mediated protein complex assembly	ISS	
GO:0050870	**involved_in** positive regulation of T cell activation	ISS	
GO:0050821	**involved_in** protein stabilization	ISS	
GO:0048291	**involved_in** isotype switching to IgG isotypes	IDA	16148103
GO:0045041	**involved_in** protein import into mitochondrial intermembrane space	IBA	21873635
GO:0044406	**NOT involved_in** adhesion of symbiont to host	IDA	20633027
GO:0043066	**involved_in** negative regulation of apoptotic process	IMP	17823127
GO:0043065	**involved_in** positive regulation of apoptotic process	IMP	17823127
GO:0043032	**involved_in** positive regulation of macrophage activation	IDA	17164250
GO:0042113	**involved_in** B cell activation	IDA	16148103
GO:0042110	**acts_upstream_of_or_within** T cell activation	IDA	15371451
GO:0042100	**involved_in** B cell proliferation	IDA	16148103
GO:0042026	**involved_in** protein refolding	IDA	11050098
GO:0034514	**involved_in** mitochondrial unfolded protein response	IBA	21873635
GO:0032755	**involved_in** positive regulation of interleukin-6 production	IDA	16148103
GO:0032735	**involved_in** positive regulation of interleukin-12 production	IDA	17164250
GO:0032733	**involved_in** positive regulation of interleukin-10 production	IDA	16148103
GO:0032729	**involved_in** positive regulation of interferon-gamma production	IBA,ISS	
GO:0032727	**involved_in** positive regulation of interferon-alpha production	IBA,IDA	17164250
GO:0009409	**involved_in** response to cold	ISS	
GO:0008637	**involved_in** apoptotic mitochondrial changes	IBA	21873635
GO:0006986	**involved_in** response to unfolded protein	IDA	11050098

Gene Ontology (GO) - Biological Process for MMP14 Gene

Filter: (41 results) See less «

GO ID	Qualified GO term	Evidence	PubMed IDs
GO:1990834	**involved_in** response to odorant	IEA	
GO:1905523	**involved_in** positive regulation of macrophage migration	IEA	
GO:1903076	**involved_in** regulation of protein localization to plasma membrane	IMP	20666777
GO:0097094	**involved_in** craniofacial suture morphogenesis	IEA	
GO:0060348	bone development	IEA	
GO:0060322	**involved_in** head development	IEA	
GO:0051895	**involved_in** negative regulation of focal adhesion assembly	IEA	
GO:0048870	**involved_in** cell motility	TAS	14645246
GO:0048771	**involved_in** tissue remodeling	IEA	
GO:0048754	**involved_in** branching morphogenesis of an epithelial tube	IEA	
GO:0048701	**involved_in** embryonic cranial skeleton morphogenesis	IEA	
GO:0045746	**involved_in** negative regulation of Notch signaling pathway	IEA,ISS	
GO:0045579	**involved_in** positive regulation of B cell differentiation	IEA,ISS	
GO:0043627	**involved_in** response to estrogen	IEA	
GO:0043615	**involved_in** astrocyte cell migration	IEA	
GO:0035988	**involved_in** chondrocyte proliferation	IEA	
GO:0035987	**involved_in** endodermal cell differentiation	IEP	23154389
GO:0031638	**involved_in** zymogen activation	IEA,IDA	20666777
GO:0030574	**involved_in** collagen catabolic process	IEA,TAS	
GO:0030335	**involved_in** positive regulation of cell migration	IDA	22065321
GO:0030324	**involved_in** lung development	IEA	
GO:0030307	**involved_in** positive regulation of cell growth	IDA	22065321
GO:0030198	**involved_in** extracellular matrix organization	IBA	21873635
GO:0022617	**involved_in** extracellular matrix disassembly	TAS	
GO:0008584	**involved_in** male gonad development	IEA	
GO:0006979	**involved_in** response to oxidative stress	IEA	
GO:0006508	**involved_in** proteolysis	IEA,TAS	14645246

Gene Ontology (GO) - Biological Process for ITGB1 Gene

Filter: adhesion (Showing 9 out of 70 results) See less «

GO ID	Qualified GO term	Evidence	PubMed IDs
GO:0007155	**involved_in** cell **adhesion**	IEA,IDA	19703720
GO:0007156	**involved_in** homophilic cell **adhesion** via plasma membrane **adhesion** molecules	TAS	10201960
GO:0007159	**involved_in** leukocyte cell-cell **adhesion**	IDA	1715889
GO:0007160	**involved_in** cell-matrix **adhesion**	IEA,IMP	18156211
GO:0007161	**acts_upstream_of_or_within** calcium-independent cell-matrix **adhesion**	IGI	19651211
GO:0031589	**involved_in** cell-substrate **adhesion**	IMP	19933311
GO:0033627	**involved_in** cell **adhesion** mediated by integrin	ISS	
GO:0033631	**involved_in** cell-cell **adhesion** mediated by integrin	IEP	17704059
GO:0034113	**involved_in** heterotypic cell-cell **adhesion**	IMP	20563599
GO:0043065	**acts_upstream_of_or_within** positive regulation of **apoptotic** process	IGI	19364818

Gene Ontology (GO) - Biological Process for TNF Gene

Filter: inflammatory *(Showing 9 out of 211 results)* See less «

GO ID	Qualified GO term	Evidence	PubMed IDs
GO:0002439	**involved_in** chronic **inflammatory** response to antigenic stimulus	IMP	14512626
GO:0002523	**involved_in** leukocyte migration involved in **inflammatory** response	IEA	
GO:0002526	**involved_in** acute **inflammatory** response	IEA	
GO:0002876	**involved_in** positive regulation of chronic **inflammatory** response to antigenic stimulus	IEA	
GO:0006954	**involved_in** **inflammatory** response	IEA,IDA	10748004
GO:0050727	regulation of **inflammatory** response	IEA	
GO:0050729	**involved_in** positive regulation of **inflammatory** response	IEA,TAS	24966471
GO:0150078	**involved_in** positive regulation of neuro**inflammatory** response	TAS	22277195
GO:1900017	**acts_upstream_of_or_within** positive regulation of cytokine production involved in **inflammatory** response	IGI	23608026
GO:1903223	**involved_in** positive regulation of **oxidative stress**-induced neuron death	IEA	
GO:0006915	**apoptotic** process	IEA	
GO:0006919	**involved_in** activation of cysteine-type endopeptidase activity involved in **apoptotic** process	IEA,IDA	16723520
GO:0008625	**involved_in** extrinsic **apoptotic** signaling pathway via death domain receptors	IEA,NAS	21319131
GO:0008630	**involved_in** intrinsic **apoptotic** signaling pathway in response to DNA damage	IEA	
GO:0043065	**involved_in** positive regulation of **apoptotic** process	IEA,IMP	9343261
GO:0043066	negative regulation of **apoptotic** process	IEA	
GO:0043154	**acts_upstream_of_or_within** negative regulation of cysteine-type endopeptidase activity involved in **apoptotic** process	IGI	31124343
GO:0043280	**involved_in** positive regulation of cysteine-type endopeptidase activity involved in **apoptotic** process	IDA	11577081
GO:0043525	**involved_in** positive regulation of neuron **apoptotic** process	IEA,ISS	
GO:0072577	**involved_in** endothelial cell **apoptotic** process	IEA	
GO:0097190	**apoptotic** signaling pathway	IEA	
GO:0097191	**involved_in** extrinsic **apoptotic** signaling pathway	IEA,IDA	11577081
GO:2000351	**involved_in** regulation of endothelial cell **apoptotic** process	IMP	10843670
GO:2001234	**acts_upstream_of_or_within** negative regulation of **apoptotic** signaling pathway	IGI	31124343
GO:2001238	**involved_in** positive regulation of extrinsic **apoptotic** signaling pathway	IBA	21873635
GO:2001240	**involved_in** negative regulation of extrinsic **apoptotic** signaling pathway in absence of ligand	IDA	10666185
GO:0034116	**involved_in** positive regulation of heterotypic cell-cell **adhesion**	IDA	10604883
GO:0045785	**involved_in** positive regulation of cell **adhesion**	IMP	23892569
GO:1904996	**involved_in** positive regulation of leukocyte **adhesion** to vascular endothelial cell	IDA	26371161
GO:1904999	**involved_in** positive regulation of leukocyte **adhesion** to arterial endothelial cell	IDA	22267480

S2 Figure: Rosavin-protein interaction analysis by blind docking

For the for proteins HSPD1, MMP14, Integrin-beta 1 subunit 1 and 2and TNF-α, we reported the top 5 poses for Rosavin-protein interaction in Figures (1-25 B). Regarding the interaction types between amino acid residues and Rosavin, grey dashed-lines and green dashed-lines indicate pi-pi stacking in parallel and perpendicular spatial respectively. Hydrogen bonds were represented in blue color and charge centers were represented by yellow spheres. The binding energy of every interaction was detailed in a bar plot representing contributing energies and potentials in Figures (1-25 A).

(A)

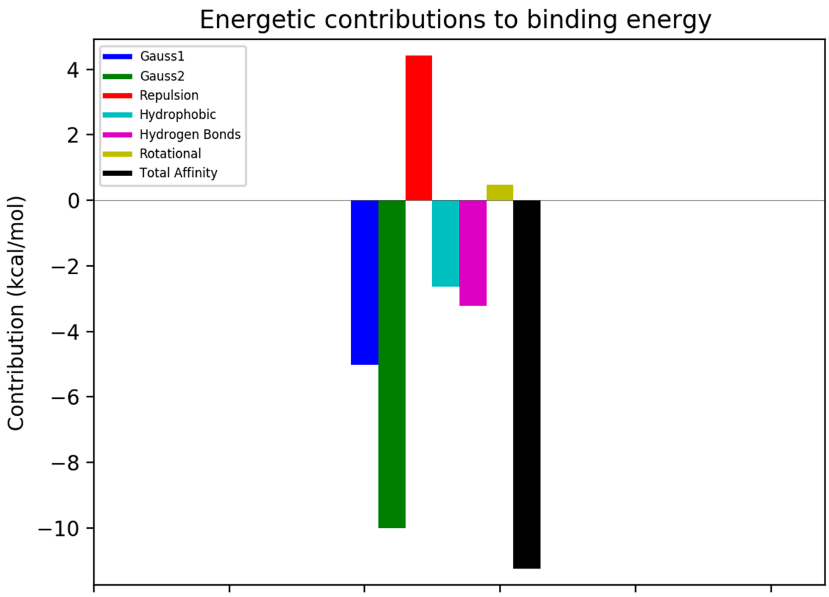

(B)

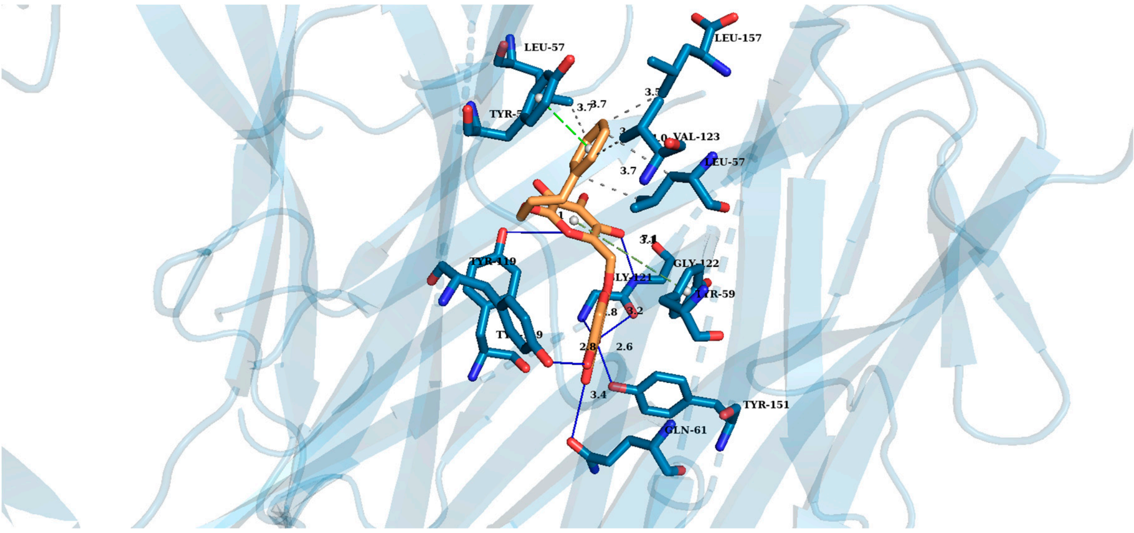

Figure S1(A) Energetic contributions of the binding energy for the first pose for Rosavin interacting with TNF-a. (B) Rosavin binding to the TNF-a first pose with binding energy of -11.50 kcal/mole.

(A)

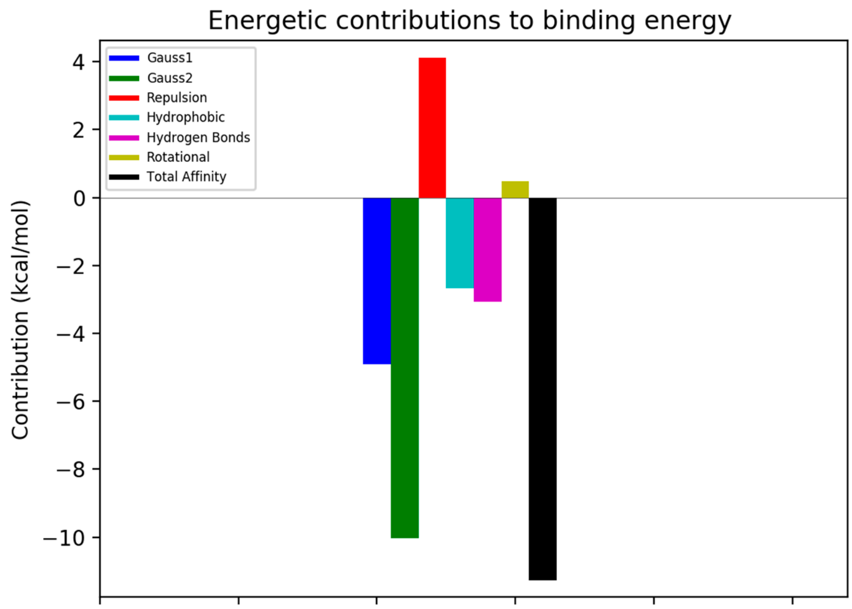

(B)

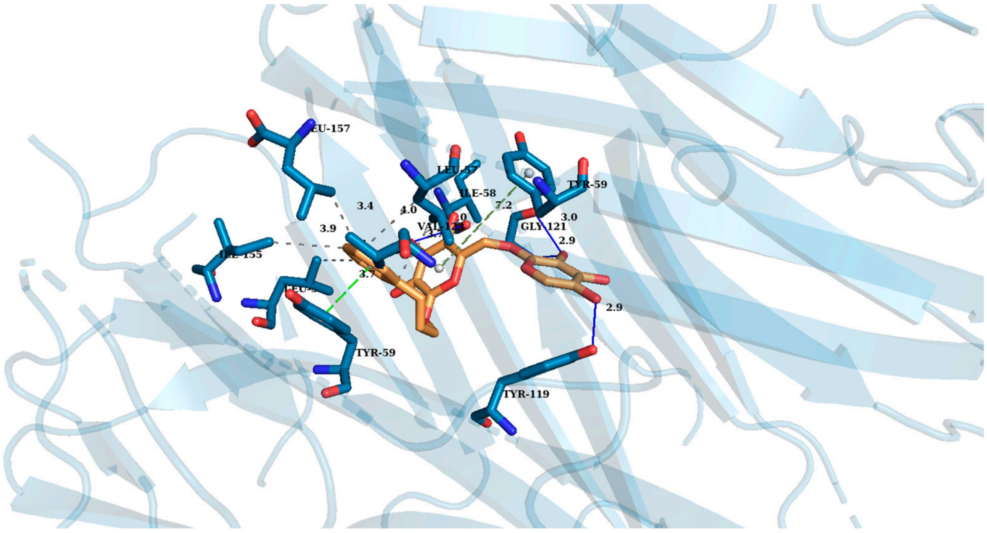

Figure S2 (A) Energetic contributions of the binding energy for the second pose for Rosavin interacting with TNF-a. (B) Rosavin binding to the TNF-a second pose with binding energy of -11.50 kcal/mole.

(A)

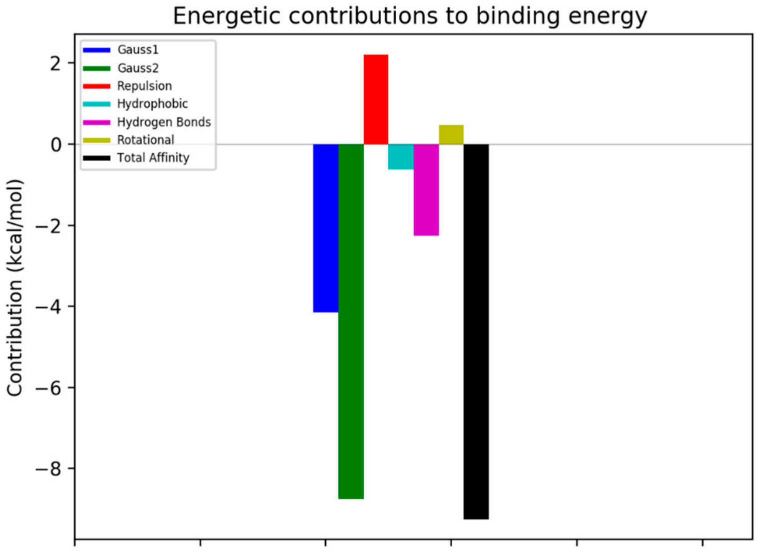

(B)

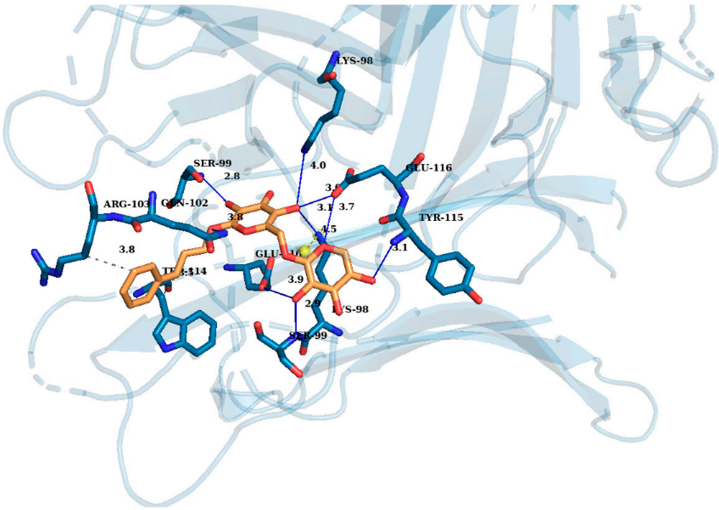

Figure S3(A) Energetic contributions of the binding energy for the Third pose for Rosavin interacting with TNF-a. (B) Rosavin binding to the TNF-a Third pose with binding energy of -9.30 kcal/mole.

(A)

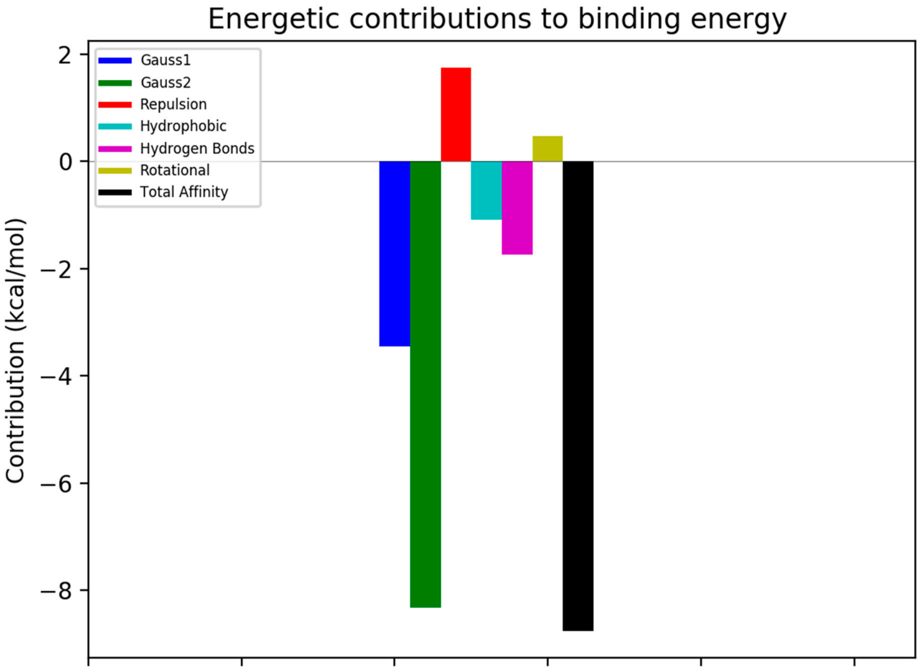

(B)

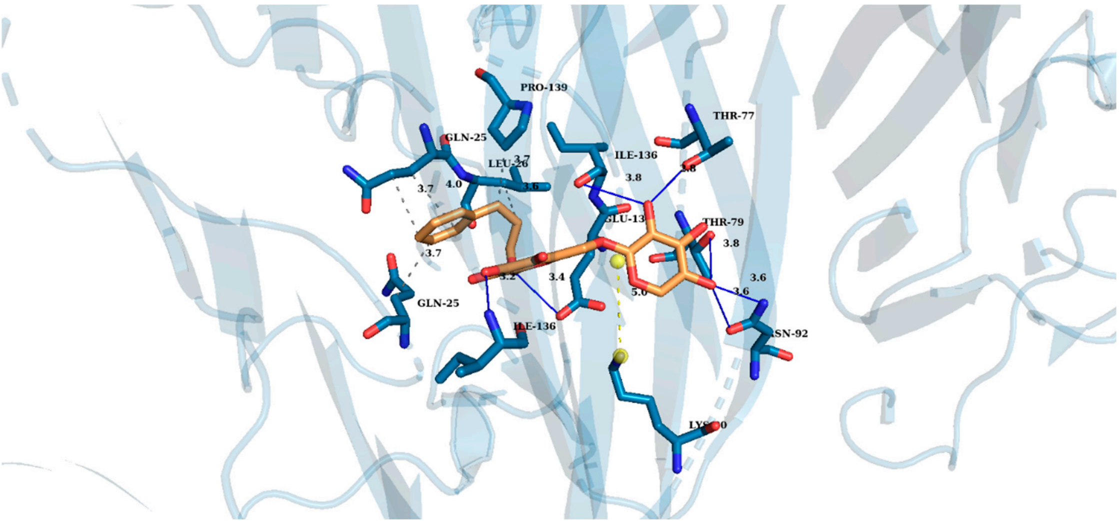

Figure S4 (A) Energetic contributions of the binding energy for the Fourth pose for Rosavin interacting with TNF-a. (B) Rosavin binding to the TNF-a Fourth pose with binding energy of -8.80 kcal/mole.

(A)

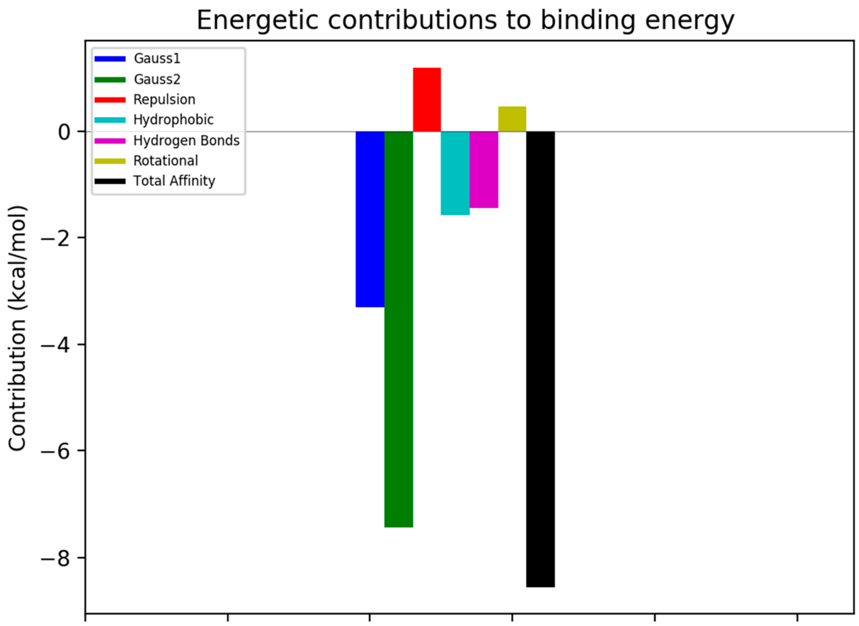

(B)

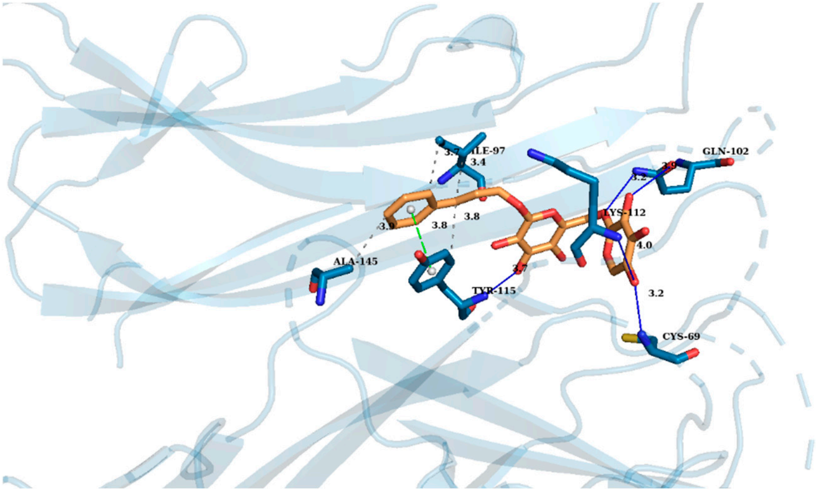

Figure S5 (A) Energetic contributions of the binding energy for the Fifth pose for Rosavin interacting with TNF-a. (B) Rosavin binding to the TNF-a Fifth pose with binding energy of -8.60 kcal/mole.

(A)

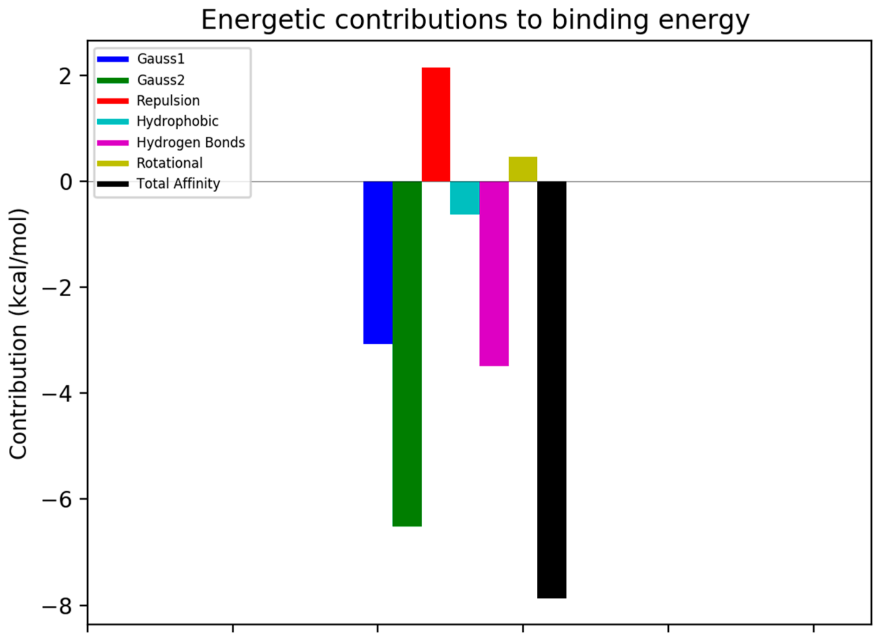

(B)

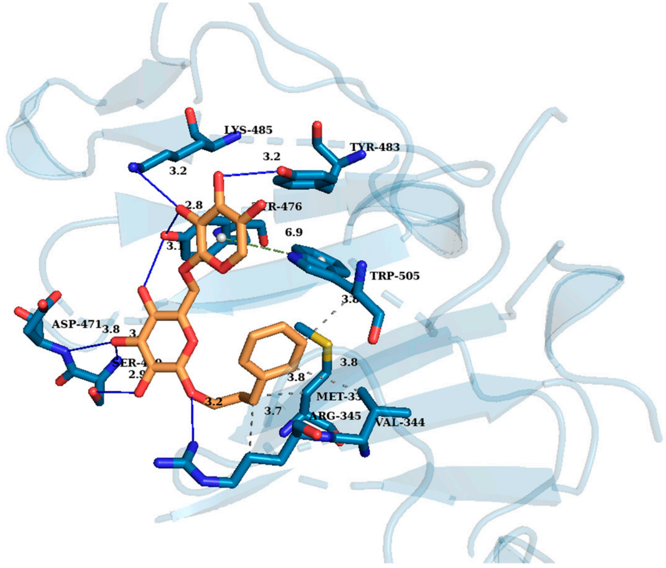

Figure S6 (A) Energetic contributions of the binding energy for the First pose for Rosavin interacting with MMP14. (B) Rosavin binding to the MT1-MMP First pose with binding energy of -7.90 kcal/mole.

(A)

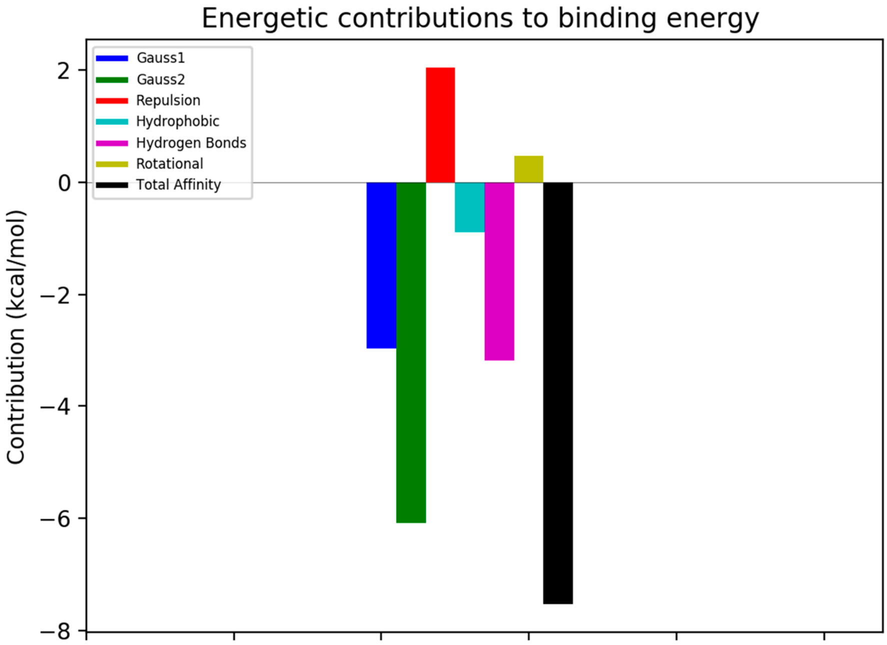

(B)

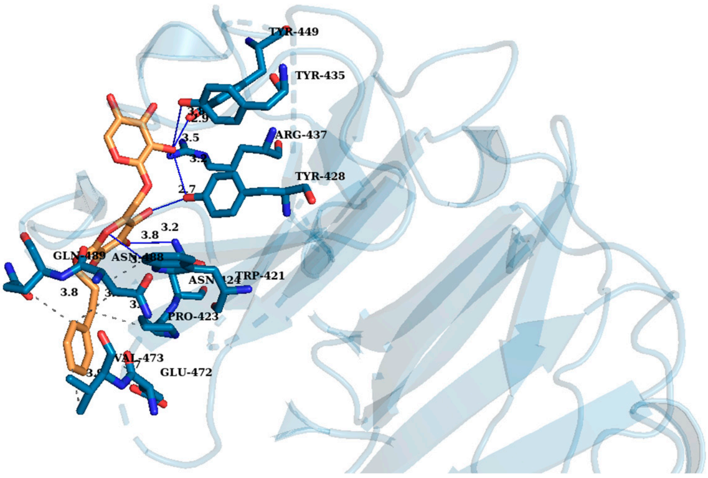

Figure S7(A) Energetic contributions of the binding energy for the Second pose for Rosavin interacting with MMP14. (B) Rosavin binding to the MT1-MMP second pose with binding energy of -7.50 kcal/mole.

(A)

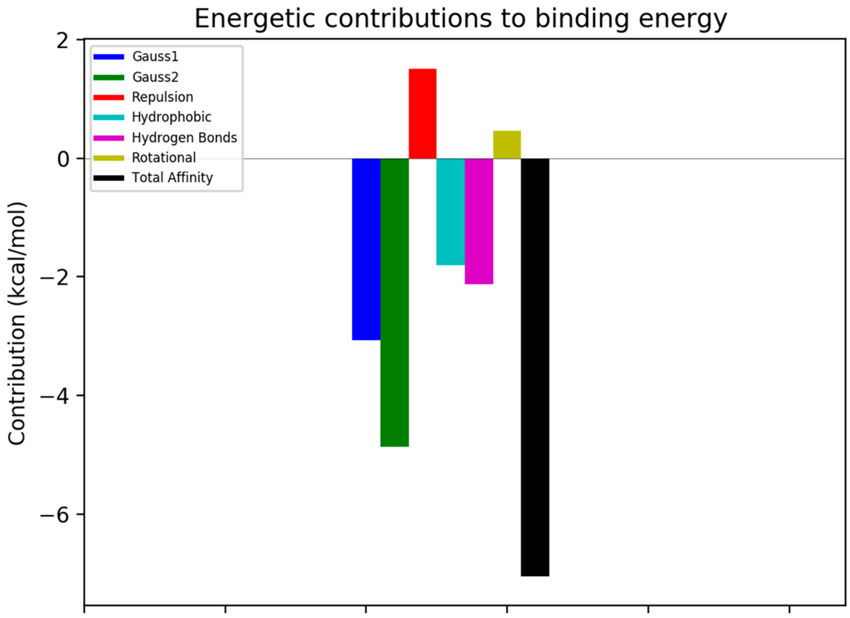

(B)

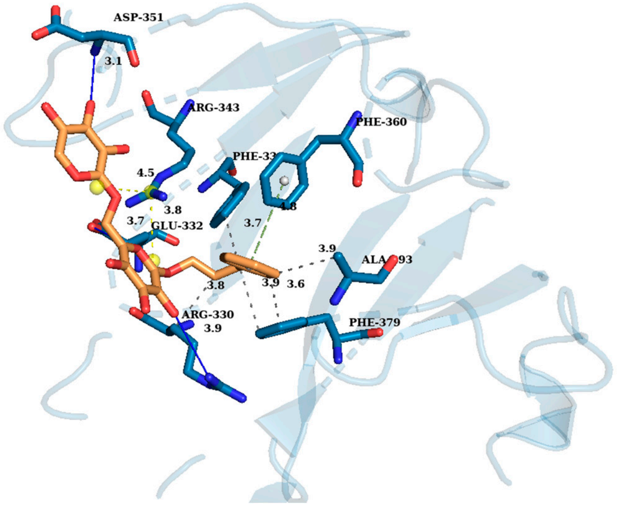

Figure S8 (A) Energetic contributions of the binding energy for the third pose for Rosavin interacting with MMP14. (B) Rosavin binding to the MT1-MMP third pose with binding energy of -7.00 kcal/mole.

(A)

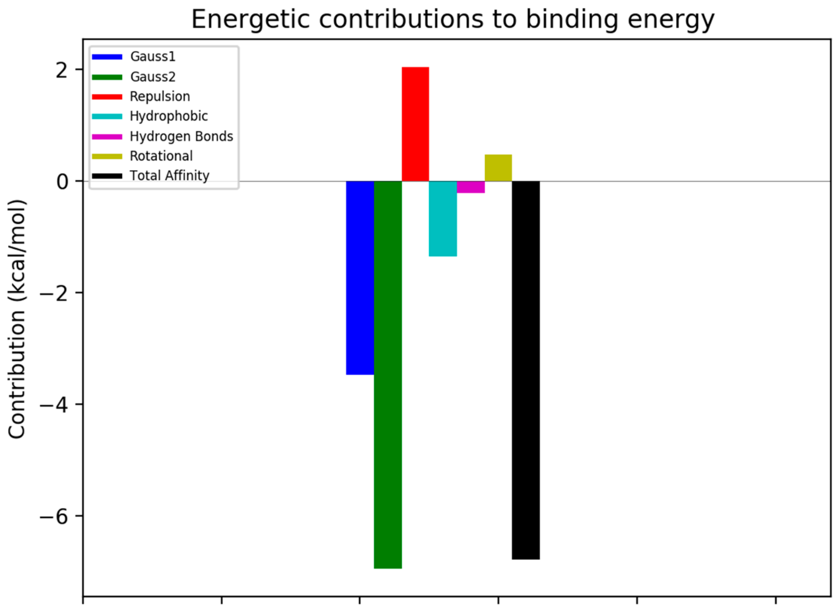

(B)

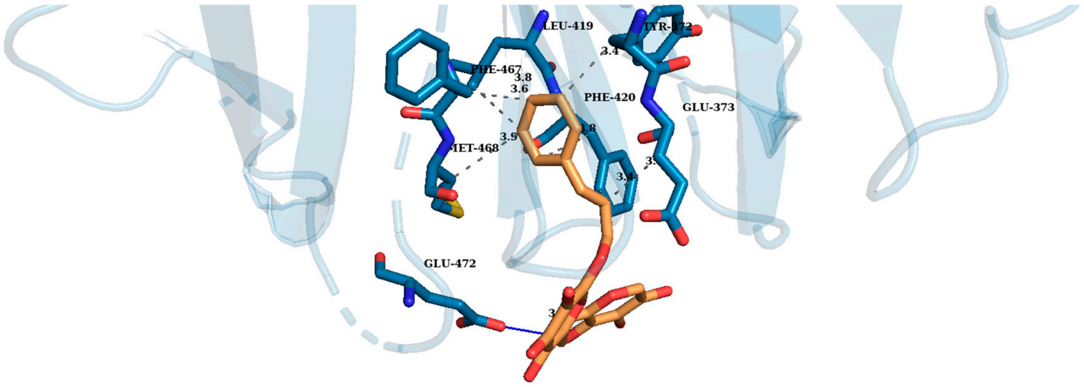

Figure S9 (A) Energetic contributions of the binding energy for the fourth pose for Rosavin interacting with MMP14. (B) Rosavin binding to the MT1-MMP fourth pose with binding energy of -6.80 kcal/mole.

(A)

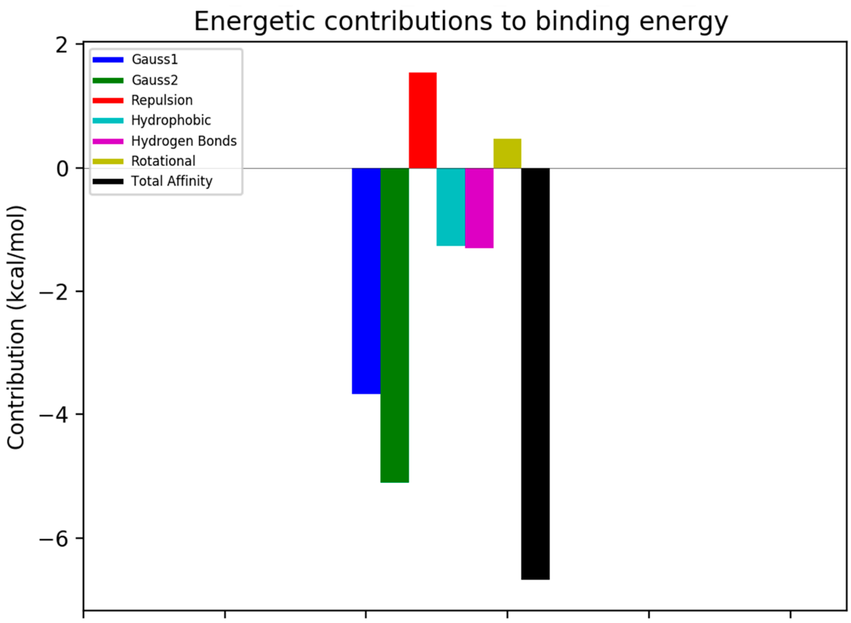

(B)

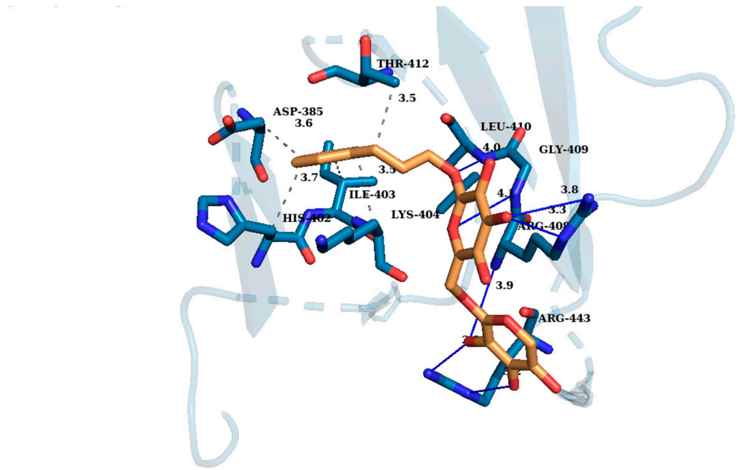

Figure S10 (A) Energetic contributions of the binding energy for the fifth pose for Rosavin interacting with MMP14. (B) Rosavin binding to the MT1-MMP fifth pose with binding energy of -6.70 kcal/mole.

(A)

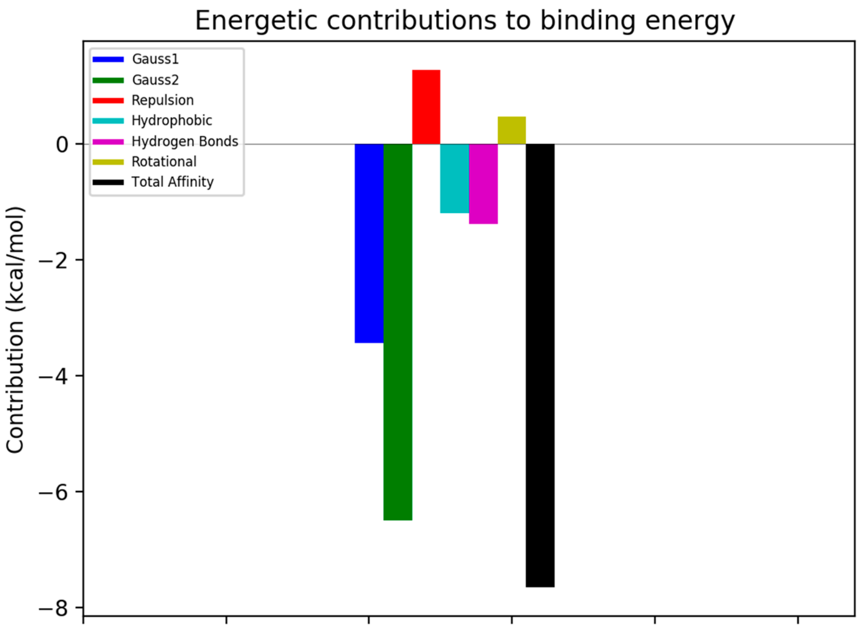

(B)

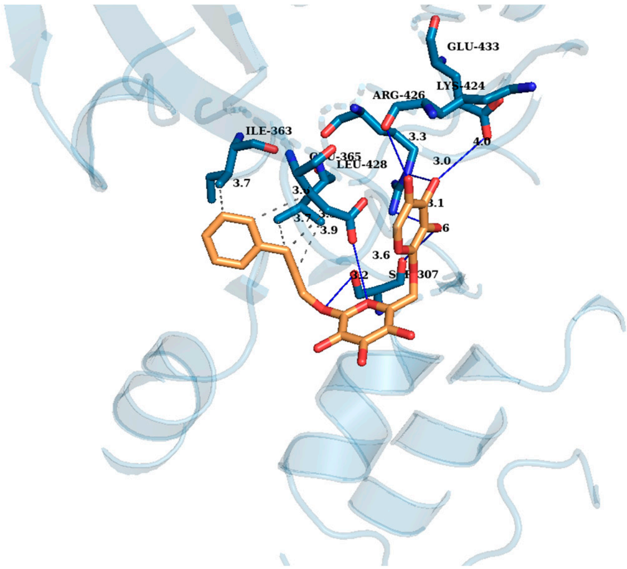

Figure S11 (A) Energetic contributions of the binding energy for the first pose for Rosavin interacting with Integrin-beta 1 Subunit (1). (B) Rosavin binding to the with Integrin-beta 1 Subunit (1) first pose with binding energy of -7.70 kcal/mole.

(A)

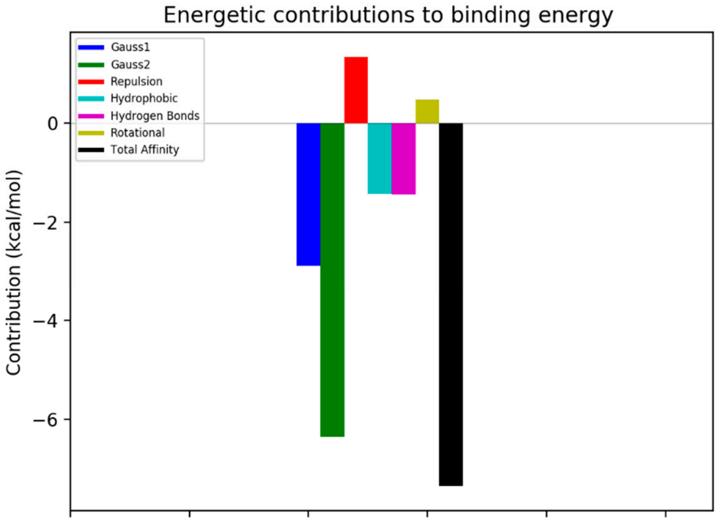

(B)

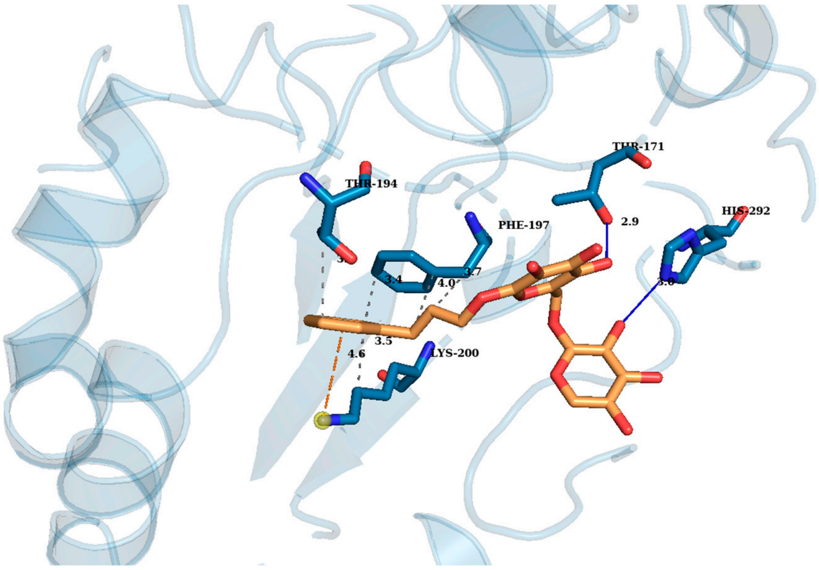

Figure S12 (A) Energetic contributions of the binding energy for the second pose for Rosavin interacting with Integrin-beta 1 Subunit (1). (B) Rosavin binding to the with Integrin-beta 1 Subunit (1) second pose with binding energy of -7.40 kcal/mole.

(A)

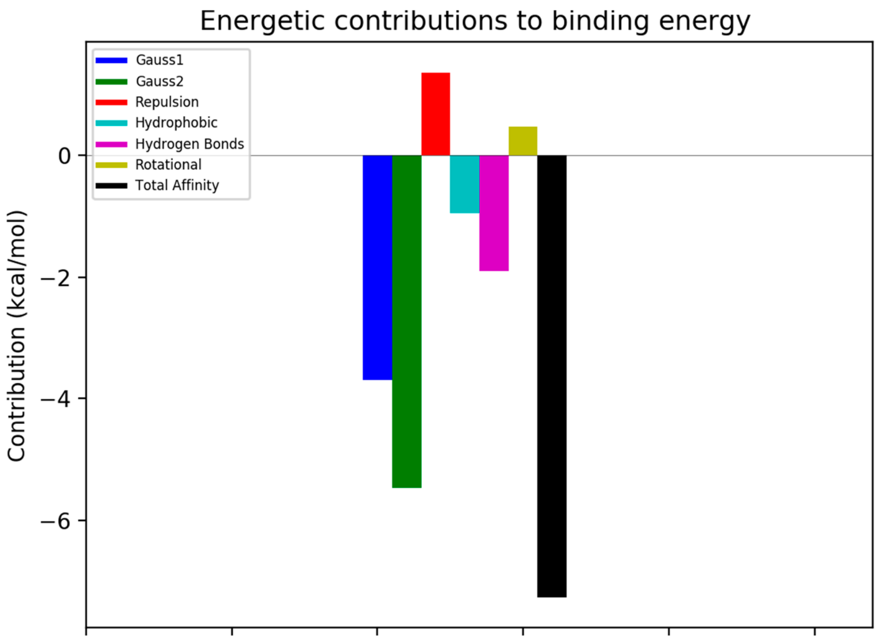

(B)

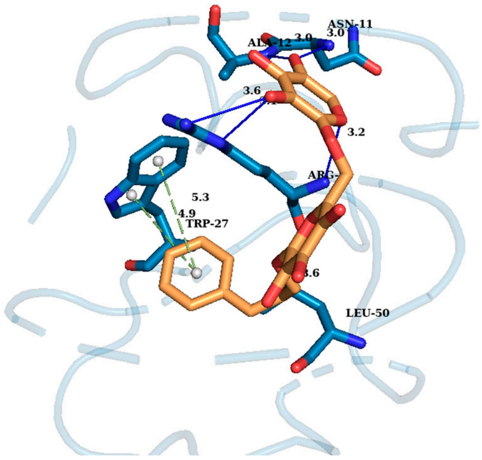

Figure S13 (A) Energetic contributions of the binding energy for the third pose for Rosavin interacting with Integrin-beta 1 Subunit (1). (B) Rosavin binding to the with Integrin-beta 1 Subunit (1) third pose with binding energy of -7.30 kcal/mole.

(A)

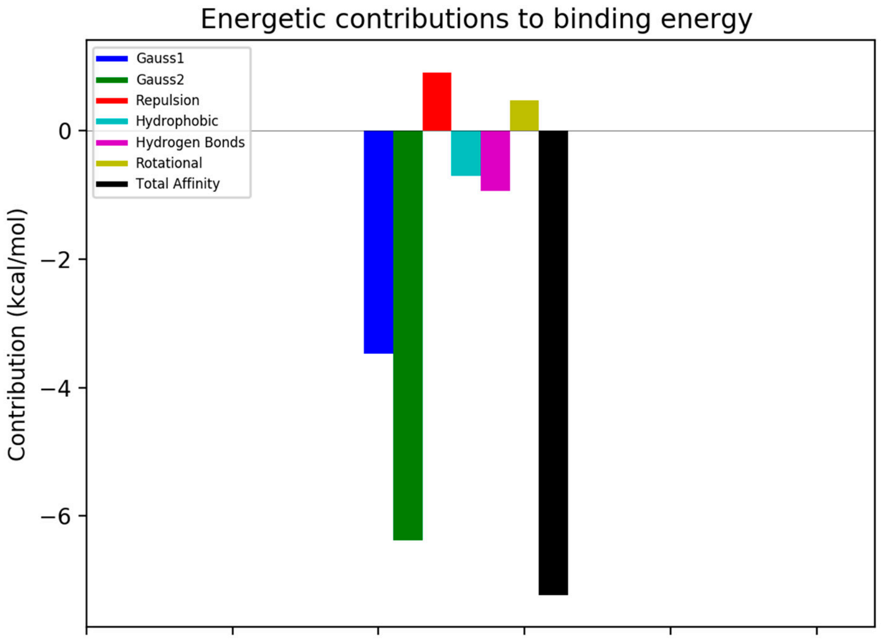

(B)

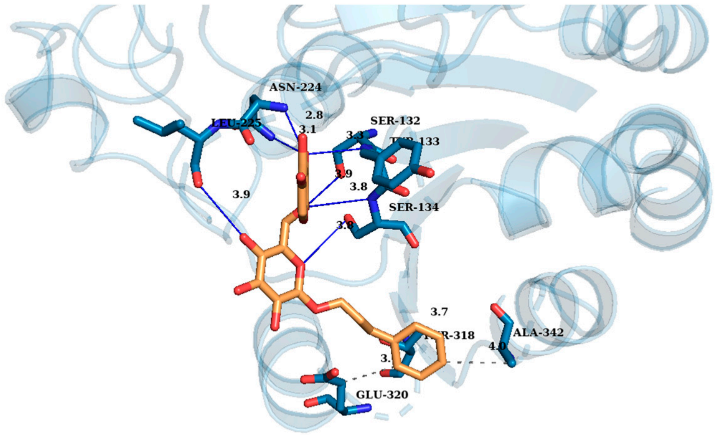

Figure S14 (A) Energetic contributions of the binding energy for the fourth pose for Rosavin interacting with Integrin-beta 1 Subunit (1). (B) Rosavin binding to the with Integrin-beta 1 Subunit (1) fourth pose with binding energy of -7.20 kcal/mole.

(A)

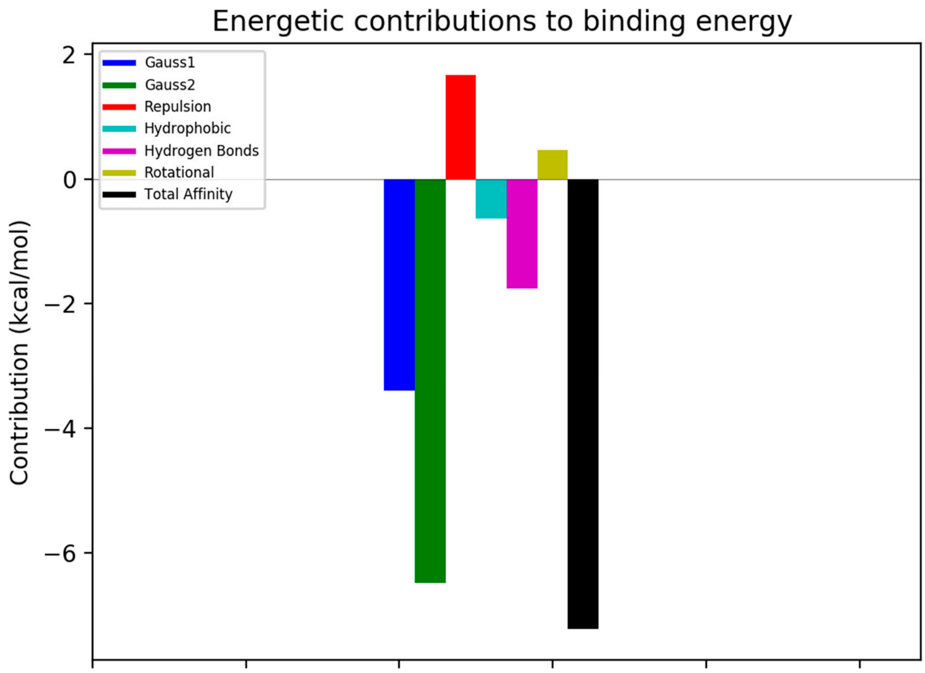

(B)

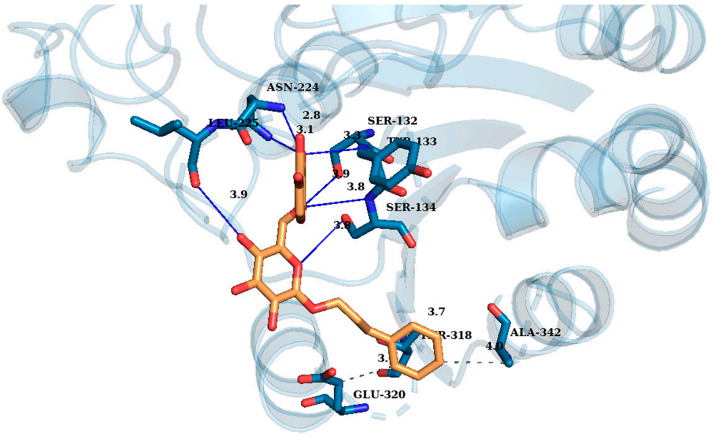

Figure S15 (A) Energetic contributions of the binding energy for the fifth pose for Rosavin interacting with Integrin-beta 1 Subunit (1). (B) Rosavin binding to the with Integrin-beta 1 Subunit (1) fifth pose with binding energy of -7.20 kcal/mole.

(A)

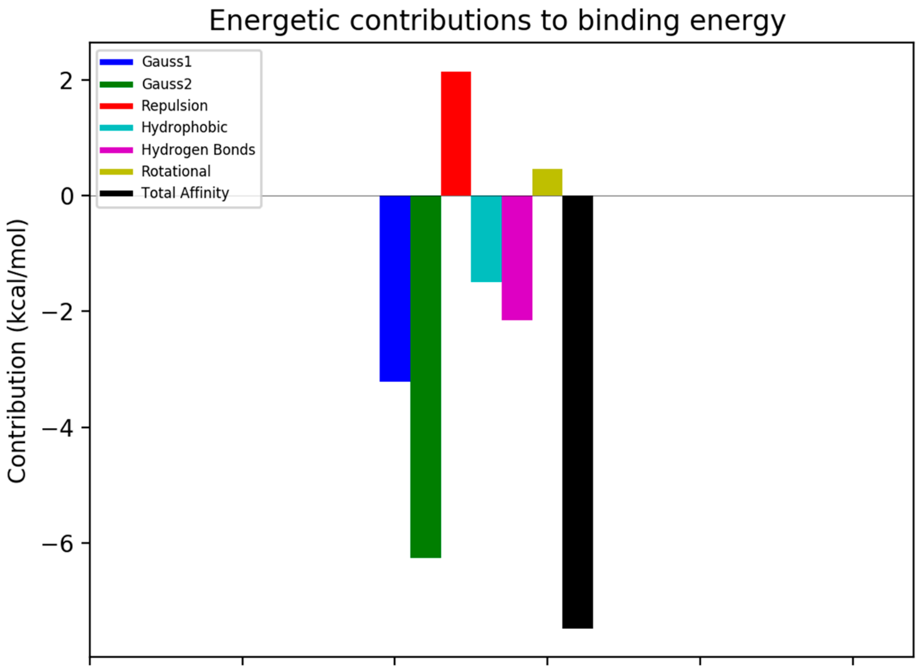

(B)

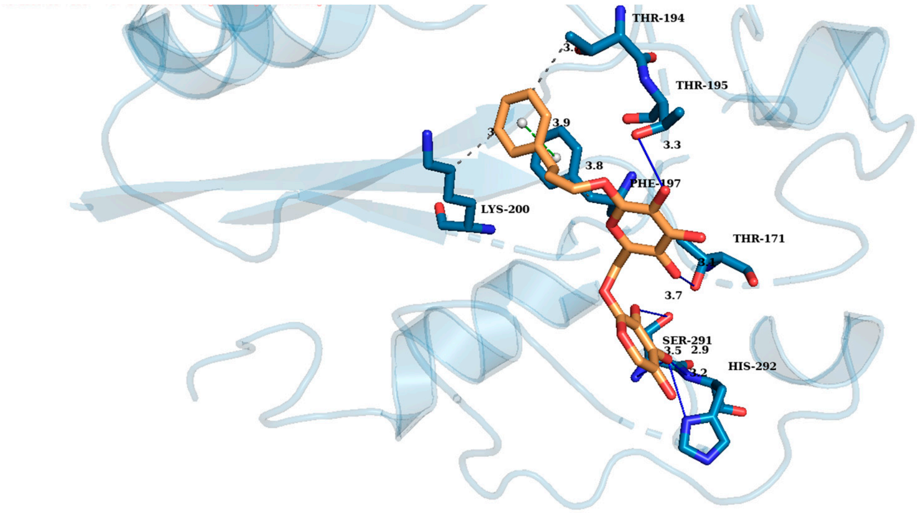

Figure S16 (A) Energetic contributions of the binding energy for the first pose for Rosavin interacting with Integrin-beta 1 Subunit (2). (B) Rosavin binding to the with Integrin-beta 1 Subunit (2) first pose with binding energy of -7.50 kcal/mole.

(A)

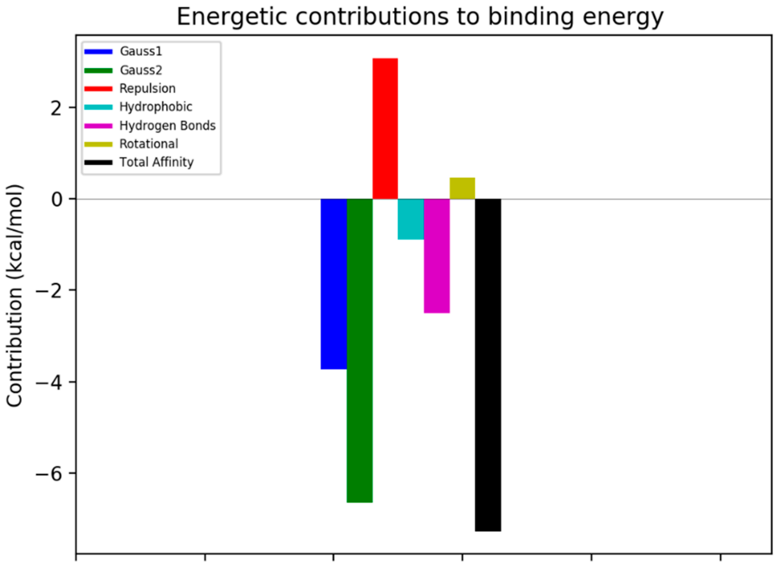

(B)

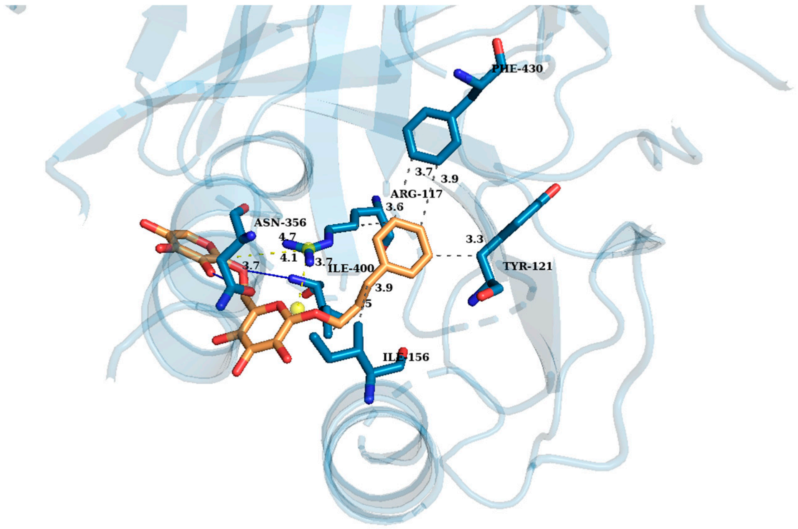

Figure S17 (A) Energetic contributions of the binding energy for the second pose for Rosavin interacting with Integrin-beta 1 Subunit (2). (B) Rosavin binding to the with Integrin-beta 1 Subunit (2) second pose with binding energy of -7.30 kcal/mole.

(A)

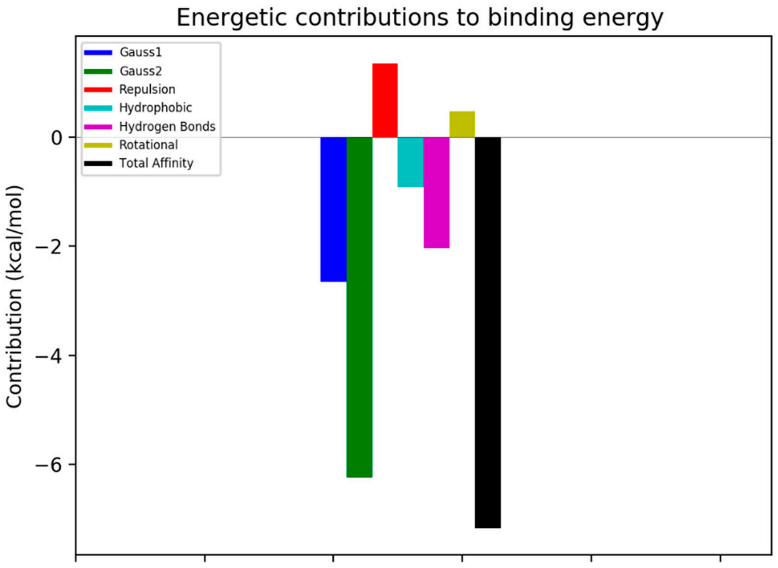

(B)

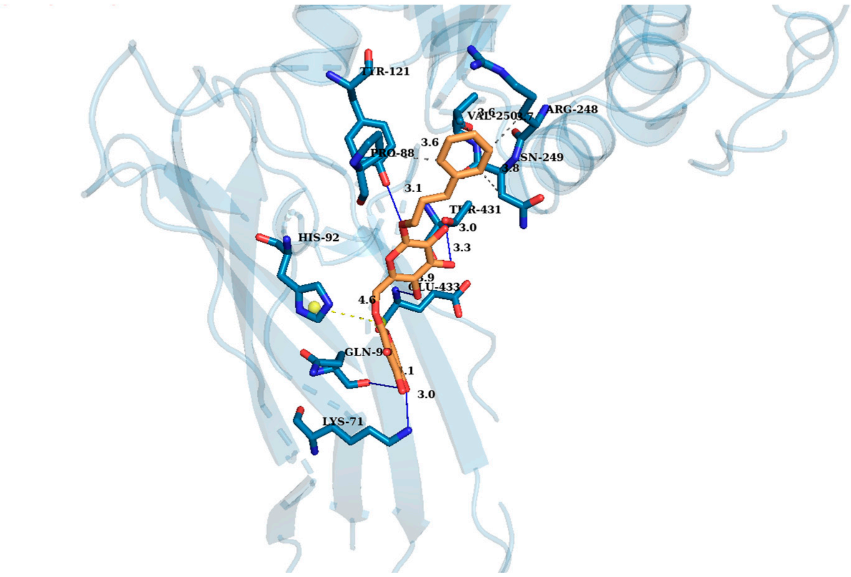

Figure S18 (A) Energetic contributions of the binding energy for the third pose for Rosavin interacting with Integrin-beta 1 Subunit (2). (B) Rosavin binding to the with Integrin-beta 1 Subunit (2) third pose with binding energy of -7.20 kcal/mole.

(A)

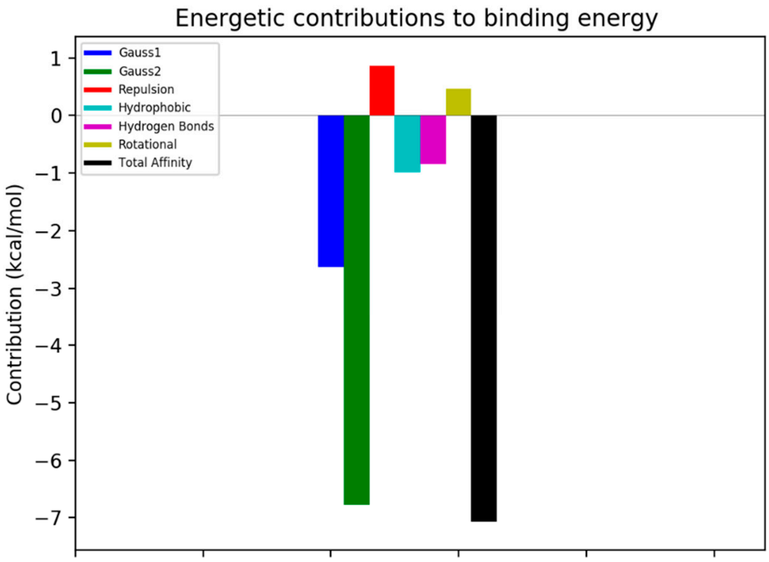

(B)

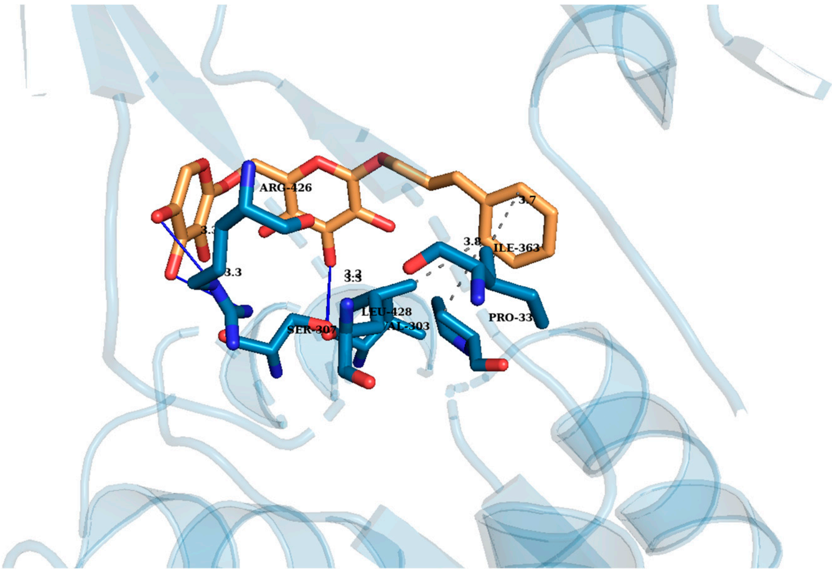

Figure S19 (A) Energetic contributions of the binding energy for the fourth pose for Rosavin interacting with Integrin-beta 1 Subunit (2). (B) Rosavin binding to the with Integrin-beta 1 Subunit (2) fourth pose with binding energy of -7.20 kcal/mole.

(A)

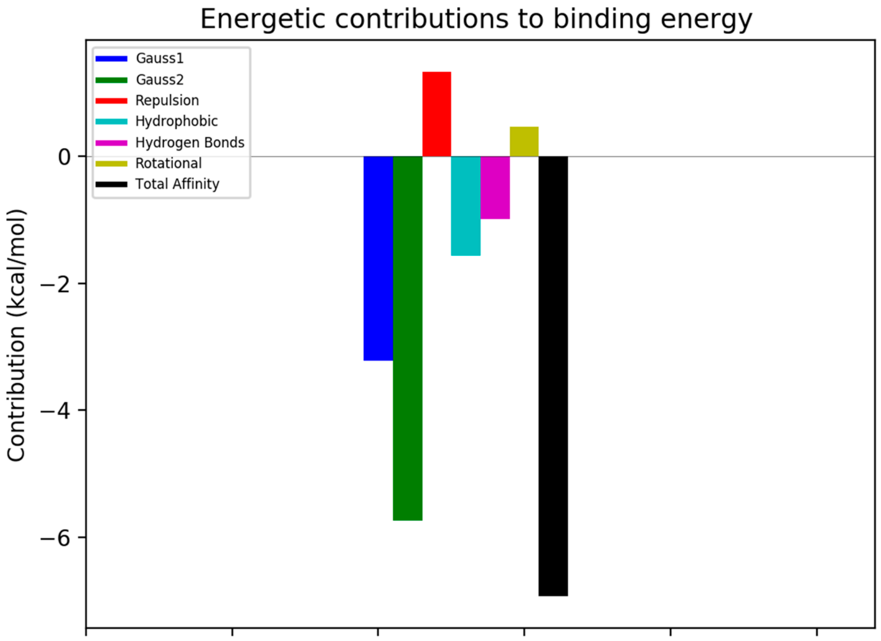

(B)

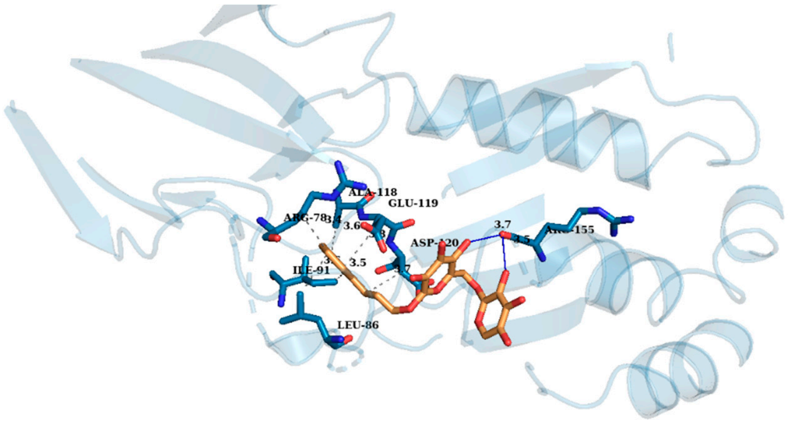

Figure S20 (A) Energetic contributions of the binding energy for the fifth pose for Rosavin interacting with Integrin-beta 1 Subunit (2). (B) Rosavin binding to the with Integrin-beta 1 Subunit (2) fifth pose with binding energy of -7.10 kcal/mole.

(A)

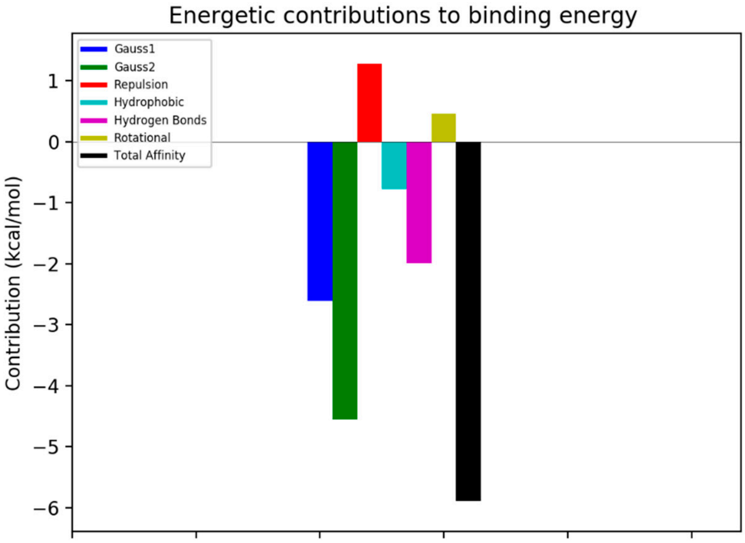

(B)

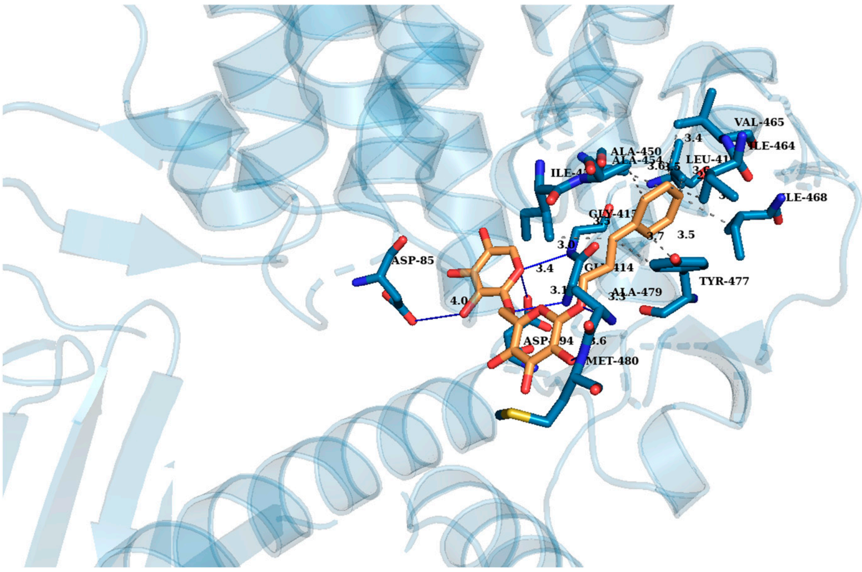

Figure S21 (A) Energetic contributions of the binding energy for the first pose for Rosavin interacting HSPD1. (B) Rosavin binding to the with HSPD1 first pose with binding energy of -8.30 kcal/mole.

(A)

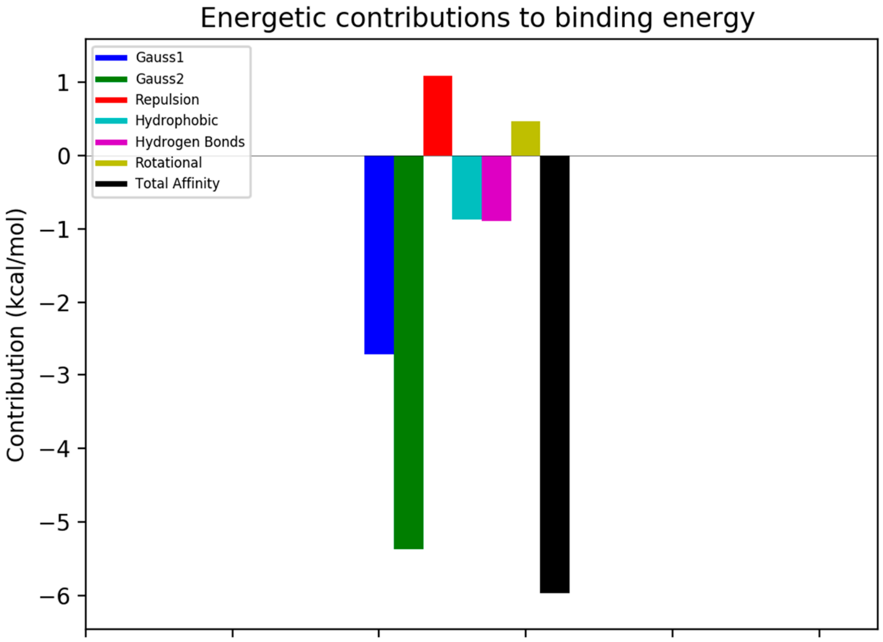

(B)

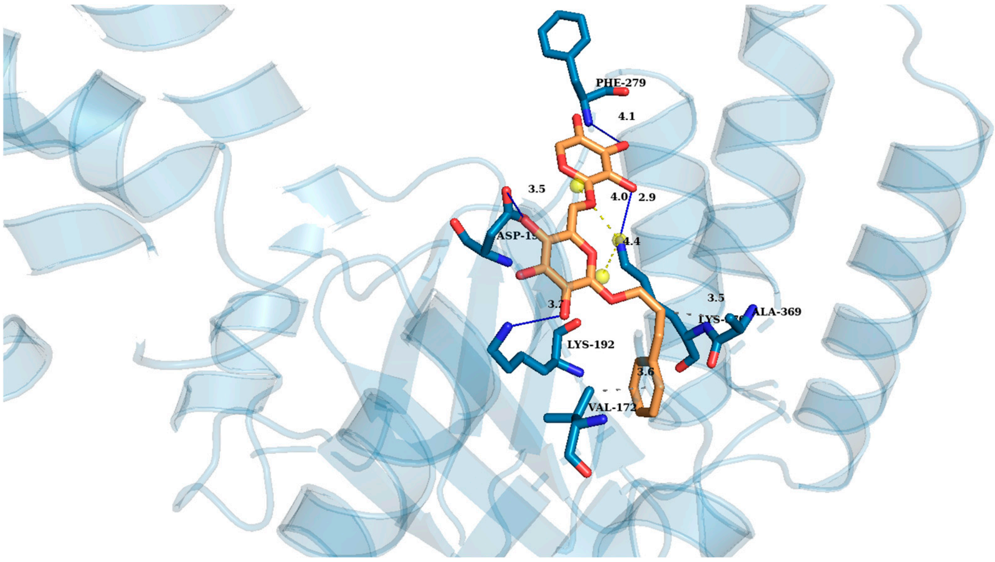

Figure S22 (A) Energetic contributions of the binding energy for the second pose for Rosavin interacting HSPD1. (B) Rosavin binding to the with HSPD1 second pose with binding energy of -7.90 kcal/mole.

(A)

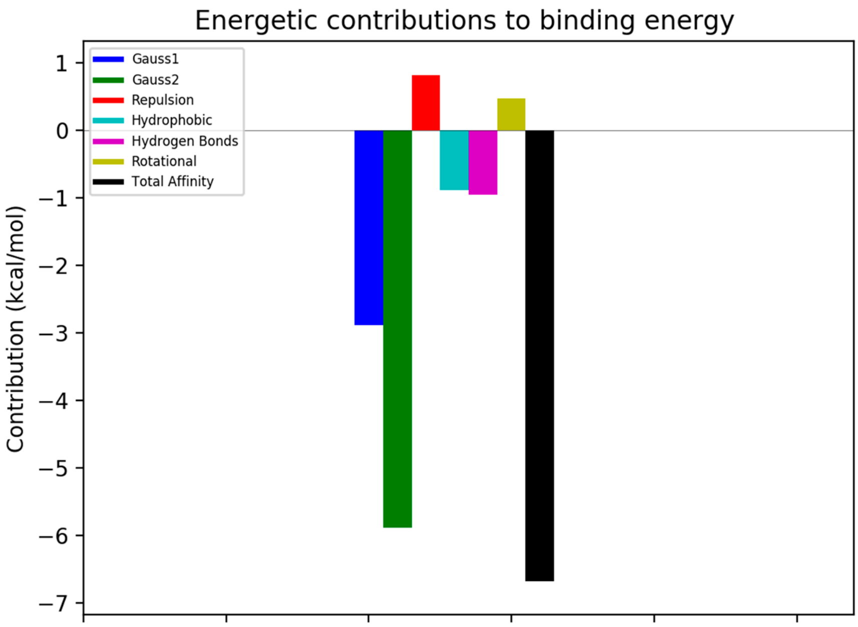

(B)

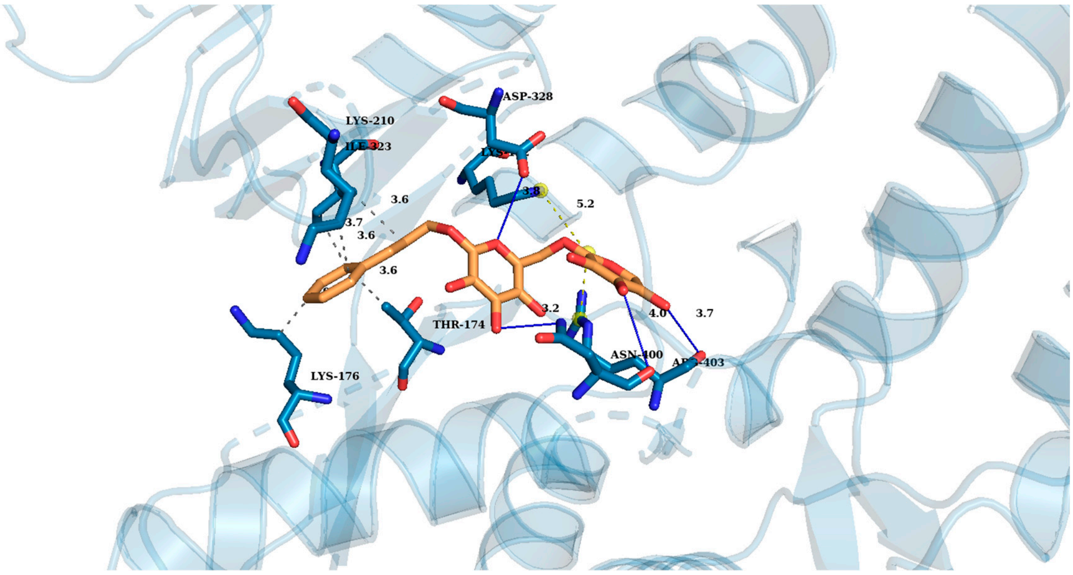

Figure S23 (A) Energetic contributions of the binding energy for the third pose for Rosavin interacting HSPD1. (B) Rosavin binding to the with HSPD1 third pose with binding energy of -6.90 kcal/mole.

(A)

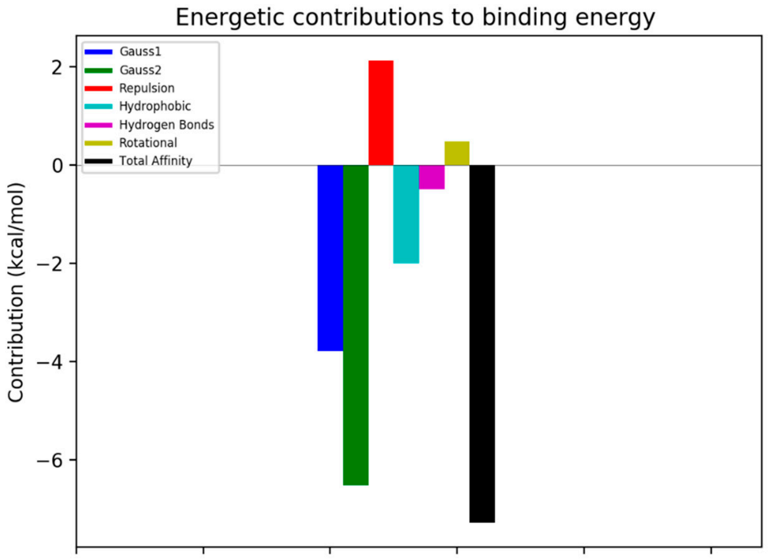

(B)

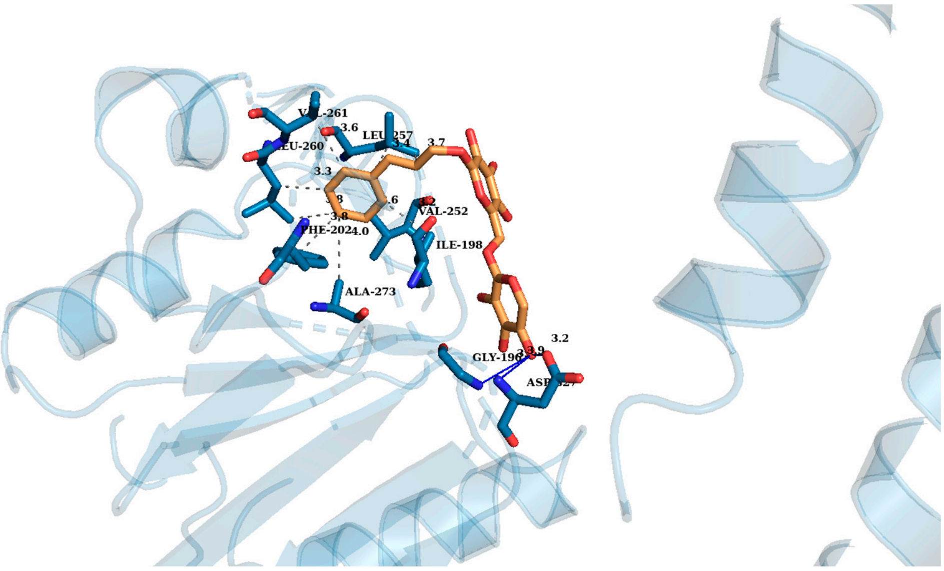

Figure S24 (A) Energetic contributions of the binding energy for the fourth pose for Rosavin interacting HSPD1. (B) Rosavin binding to the with HSPD1 fourth pose with binding energy of -7.30 kcal/mole.

(A)

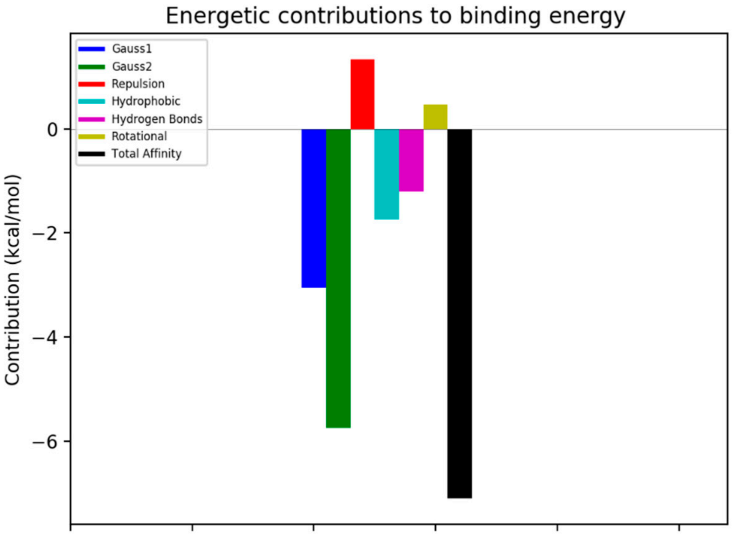

(B)

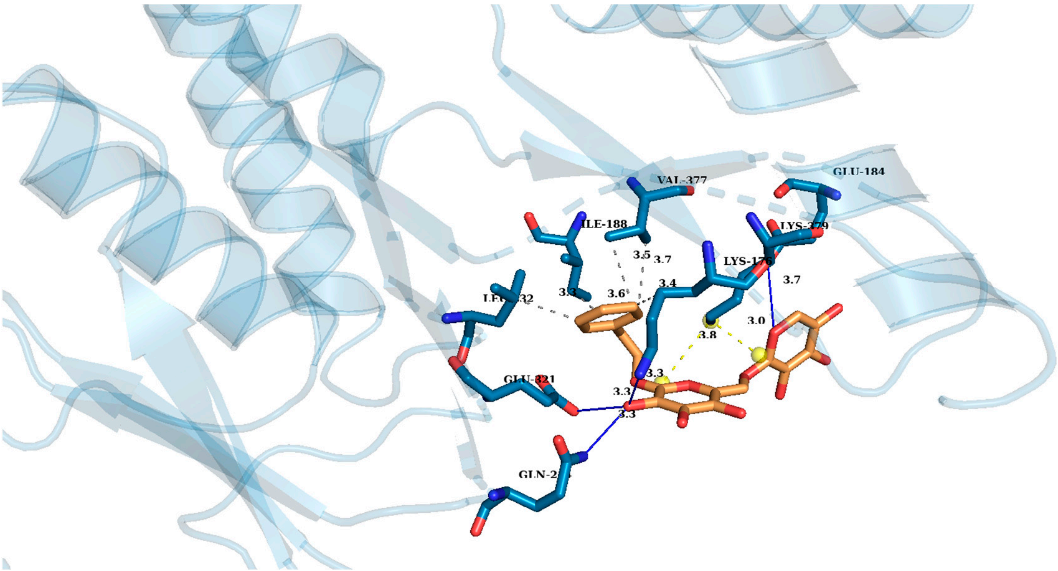

Figure S25 (A) Energetic contributions of the binding energy for the fifth pose for Rosavin interacting HSPD1. (B) Rosavin binding to the with HSPD1 fifth pose with binding energy of -7.10 kcal/mole.

S3 Figure: Rosavin-miRNA-6881-5p analysis

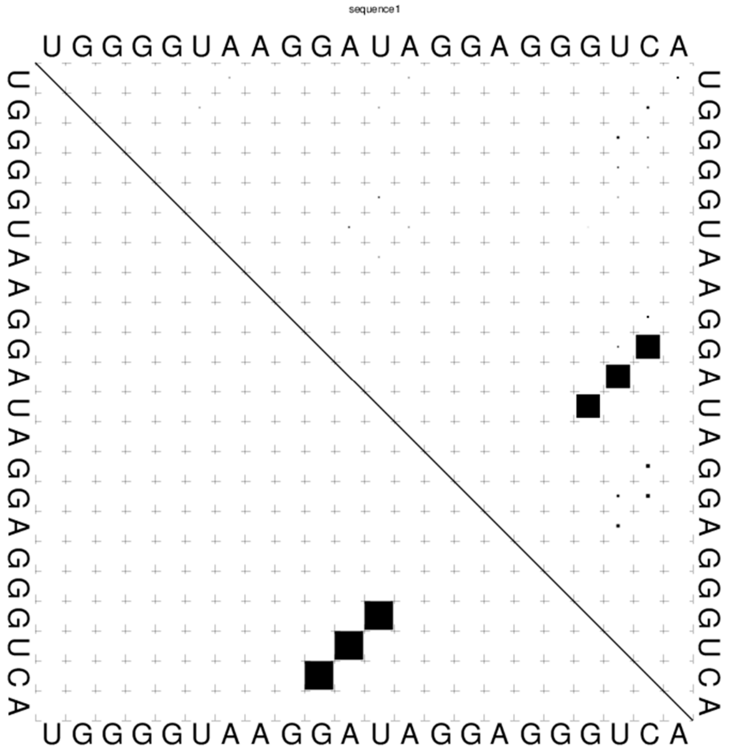

Figure S26 miR-6881-5p secondary structure probability dot plot

Rosavin miR-6881-5p interaction is indicated in the following Figures (Figures 26-31) where green interaction indicates hydrogen bonding, orange interaction indicates pi-anion interaction, pink interaction indicates pi-alkyl interaction and light blue interaction indicates pi-hydrogen interaction. The length of bonds is computed and presented in angstroms.

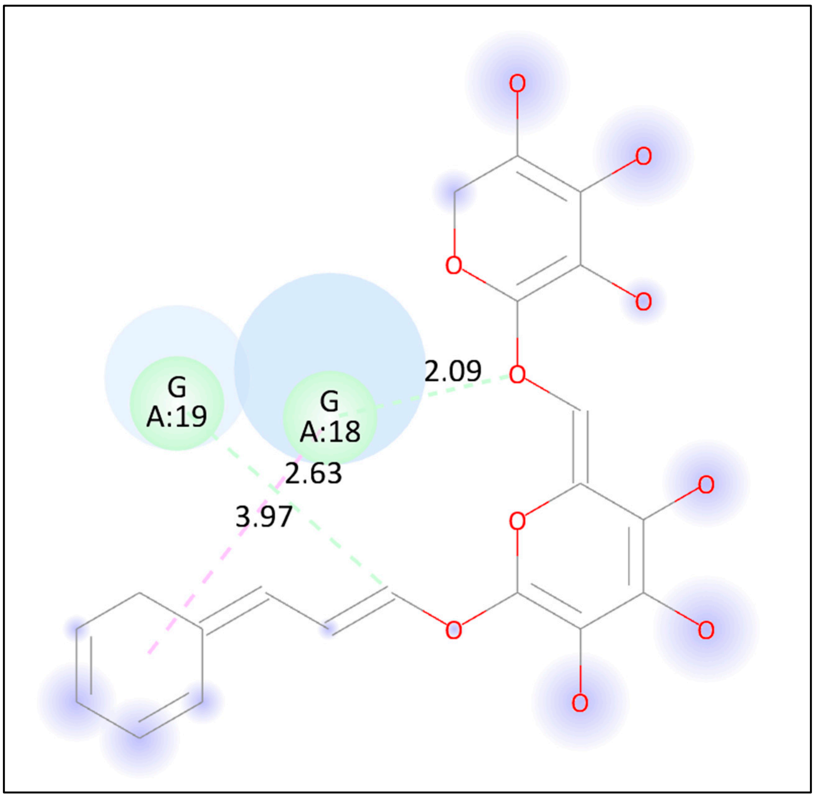

Figure S27 First pose of miR-6881-5p Rosavin interactions

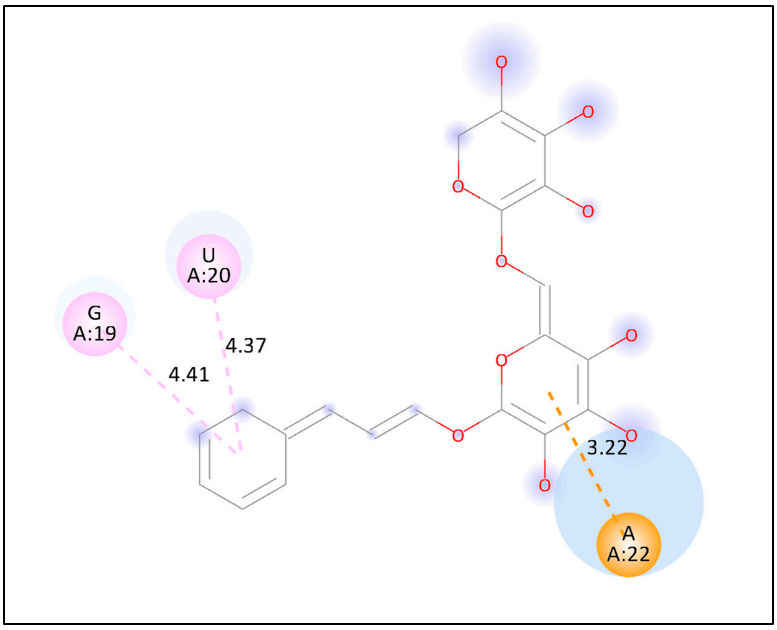

Figure S28 Second pose of miR-6881-5p Rosavin interactions

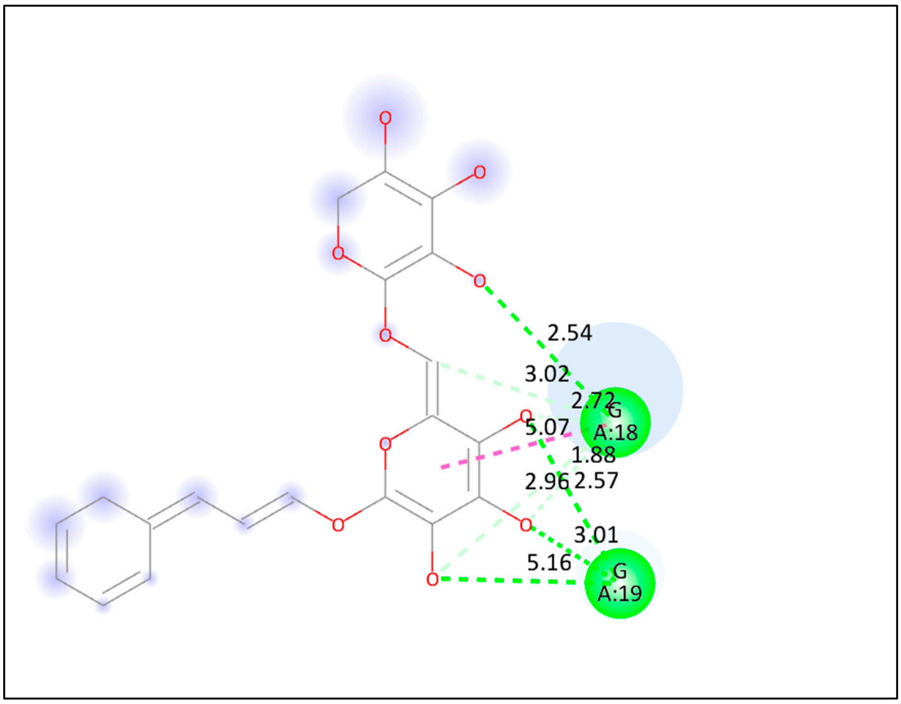

Figure S29 Third pose of miR-6881-5p Rosavin interactions

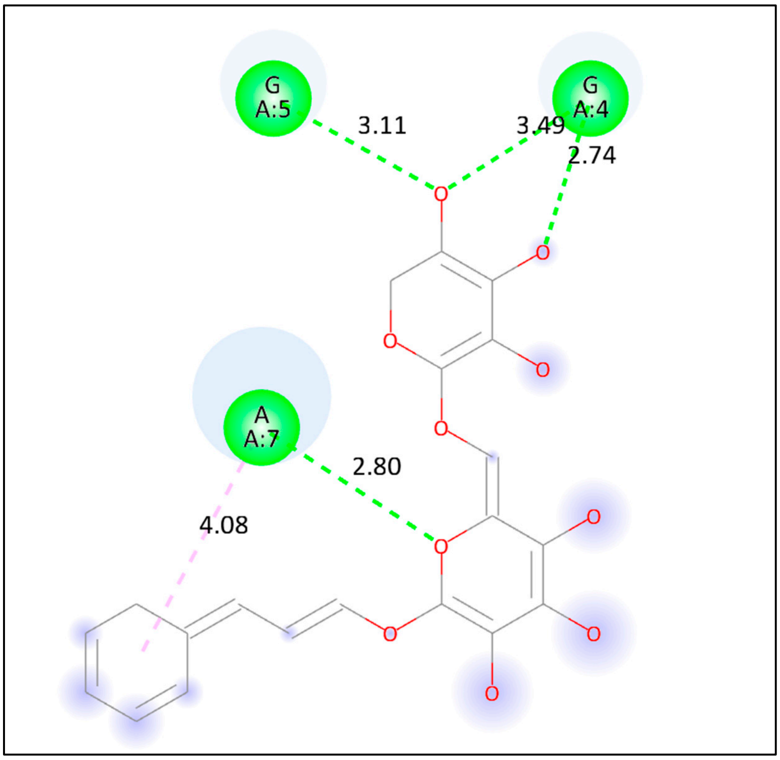

Figure S30 Fourth pose of miR-6881-5p Rosavin interactions

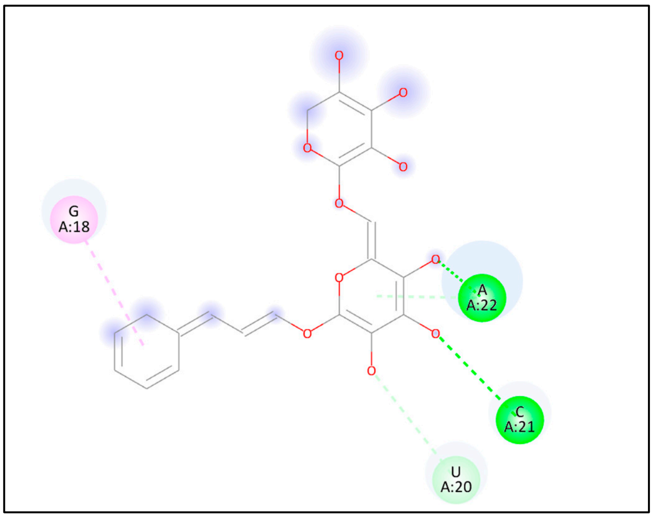

Figure S31 Fifth pose of miR-6881-5p Rosavin interactions

Supplementary tables

Table S1. : List of primer assays.

**Primer Assay**	**GeneGlobe ID**
HSPD1 (XR_037196)	QT01670291
MMP14 (NM_004995)	QT00001533
ITGB1 (NM_004763)	QT00024654
GAPDH (NM_001256799)	QT00079247
TNF (NM_000594)	QT00029162
miR-6881-5p_1	MS00048069
SNORD72_11	MS00033719
SPARCL1-1:2 (ENST00000506480)	SBH0300480
GAD1_1390172	SBH0146388

Table S2: Rosavin miR-6881-5p binding energy details (Energy in kcal/mole)

Poses	Total E	LJ	Electrostatic	Pol	Ligand E	SASA	H-bond	RE	LJ ligand
1	-39.406	-22.67	-0.673	-0.389	0.594	26.429	-18.546	1.05	-2.287
2	-38.743	-23.30	-0.513	-0.286	0.394	29.103	-16.237	0.924	-2.287
3	-38.709	-21.59	0.223	-0.882	0.573	23.333	-12.868	1.06	-2.287
4	-38.500	-23.47	0.211	-0.879	0.566	28.924	-12.945	0.939	-2.287
5	-34.512	-21.00	0.511	-0.871	0.456	26.688	-20.415	0.801	-2.287

Total E: Total Binding Energy

LJ: Lennard-Jones potential energy

Pol: Polarization Energy

Ligand E: Self-energy of ligand

SASA: solvent-accessible surface area potential

RE: Self-energy of receptor

LJ ligand: Internal Lennard-Jones Energy
